# Development of Dilated Cardiomyopathy in a Young Woman After Bilateral Cardiac Sympathetic Denervation in Catecholaminergic Polymorphic Ventricular Tachycardia

**DOI:** 10.7759/cureus.32406

**Published:** 2022-12-11

**Authors:** Karolina Janiec, Radoslaw Krawczykiewicz

**Affiliations:** 1 Department of Cardiology, Specialist Hospital in Stalowa Wola, Stalowa Wola, POL

**Keywords:** sudden cardiac death (scd), left ventricular systolic dysfunction, bigeminy, systolic heart failure, heart failure with reduced ejection fraction, implantable cardioverter-defibrillator (icd), cardiomyopathy, bilateral cardiac sympathetic denervation, catecholaminergic polymorphic ventricular tachycardia, dilated cardiomyopathy (dcm)

## Abstract

Catecholaminergic polymorphic ventricular tachycardia (CPVT) can cause fatal tachyarrhythmias brought on by physical or emotional stress. Previously published cases deny the association between CPVT with dilated cardiomyopathy (DCM). However, rare cases show a possible association between CPVT and DCM development. Our case is one of them; we describe the development of dilated cardiomyopathy in a young woman who, six years earlier, went through bilateral cardiac sympathetic denervation (CSD) because of symptomatic catecholaminergic polymorphic ventricular tachycardia.

## Introduction

Dilated cardiomyopathy (DCM) is the most common form of cardiomyopathy, it typically affects young adults and is characterized by developing left ventricular wall thinning and distention which cause progressive impairment of heart function. The diagnosis of DCM is identified most commonly by echocardiography (ECHO). DCM may result from different underlying conditions; the cause is not identified in most patients. However, most of the previous studies described possible inflammatory and genetic factors. Familial DCM is characterized by a broad spectrum of genetic etiologies. One of the key components of familial DCM is its possible association with arrhythmias and skeletal myopathies [1].

Catecholaminergic polymorphic ventricular tachycardia (CPVT) is a lethal genetic arrhythmia that usually develops in young people without known heart disease. Lack of structural heart abnormality is a key feature of CPVT. Association between CPVT and DCM has not been determined. However, there is a growing body of literature that DCM can complicate the clinical course of CPVT. The key determinant of CPVT is syncopal episodes arising due to physical stress like exercises or acute emotional stress. Sympathetic nervous system activation plays a crucial role in the pathogenesis of CPVT. Resulting polymorphic ventricular tachycardia (VT) can progress quickly into ventricular fibrillation (VF) and cause sudden cardiac death (SCD) if defibrillation is not easily available [2].

## Case presentation

A 36-year-old Caucasian female with a history of bilateral cardiac sympathetic denervation (CSD) due to CPVT presented in 2021 to our rural community hospital with episodic atypical chest pain in stressful situations, heart palpitations, fatigue, and dyspnea on exertion for the last month.

In 2015 the patient suffered sudden cardiac arrest, which occurred at her brother’s funeral - she was successfully resuscitated and admitted to our hospital. During further hospitalization, the patient required five defibrillations due to VF recurrence. Serial electrocardiograms (EKGs) exposed no signs of short QT, long QT, or Brugada syndrome. Telemetry and Holter monitor recordings documented bidirectional or polymorphic ventricular tachycardia (bidVT/pVT) runs [3]. The Transthoracic ECHO and coronary angiography were normal at that time. Based on the received data, a decision about implantable cardioverter defibrillator (ICD) implantation was established. After the procedure patient felt good, there were no following ICD interventions, and she was discharged from the hospital on metoprolol (50 mg PO twice daily). Additionally genetic and family screenings were recommended. After two years, multiple ICD shocks were registered due to VF episodes caused by emotional stress. After additional workup and consultation with cardiologists specialized in the field of electrophysiology, the decision about sympathetic ganglia and fibers removal from the level of Th1 to Th5 was agreed upon. The patient was referred to the regional thoracic surgery division, and bilateral CSD was performed [3]. Previous studies have demonstrated that beta-blockers, calcium channel blockers, and/or flecainide are first-line therapy used in CPVT. In refractory cases, ICD and sympathetic denervation are recommended [4]. The limited access to subcutaneous ICDs, genetic testing, and flecainide, affects the quality of care in patients with CPVT and other inherited arrhythmogenic diseases in Poland. There were no ICD interventions during follow-up visits [3,5]. The patient’s brother died at a young age, and sudden cardiac death was suspected, but the exact cause of his death wasn’t established. According to the patient’s coverage, her distant cousin also had cardiac arrhythmia at a young age which required ICD implantation. Genetic testing can help identify the mutation causing CPVT, but the patient didn’t follow this recommendation. Her 12-year-old daughter was the only screened family member, and no abnormalities were found.

In 2021 patient was readmitted to our hospital because she complained of episodic atypical chest pain in stressful situations, feeling of heart palpitations, fatigue, and dyspnea on exertion for the last month. Physical examination showed a normal body temperature and normal oxygen saturation at room air. Her mucous membranes appeared pale. Cardiovascular examination revealed blood pressure of 113/73 mmHg, a regular heart rate of 60/min, and on auscultation, a holosystolic murmur was heard at the apex. Pulmonary, abdominal, and neurological examinations were unremarkable.

**Figure 1:**
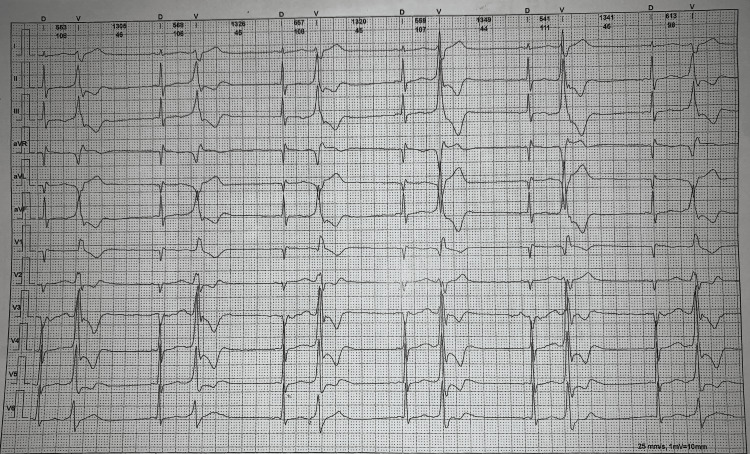
Patient's ECG: Ventricular Bigeminy

An electrocardiogram (ECG) showed a regular sinus rhythm of 64 bpm, and all the important intervals on this recording were within normal ranges. Holter’s study (Figure 1) demonstrated normal sinus rhythm with a heart rate range of 48 bpm during sleep to 77 bpm; the average heart rate was 55 bpm. There were no pauses seen. There were 855 isolated polymorphic premature ventricular contractions, 97 runs of ventricular bigeminy, and four runs of ventricular trigeminy. Chest X-ray (CXR) was normal.

Laboratory evaluation on admission revealed an iron deficiency hypochromic microcytic anemia: hemoglobin (Hb) of 9.6 g/dl [Normal (N) = 11.2-15.7], mean corpuscular volume (MCV) of 67.2 fL (N = 79.4-94.8), mean corpuscular hemoglobin (MCH) of 20.2 pg (N = 25.6-32.2), mean corpuscular hemoglobin concentration (MCHC) of 30 g/dL (N = 32.2-35.5), serum N-terminal pro-brain natriuretic peptide (NT-pro-BNP) level of 104.3 pg/ml (N = 0-125), D-dimers of 264 ng/ml (N = 0-500) and a ferritin level of 9.76 ng/ml (N = 13-150). The electrolytes were normal.

**Figure 2:**
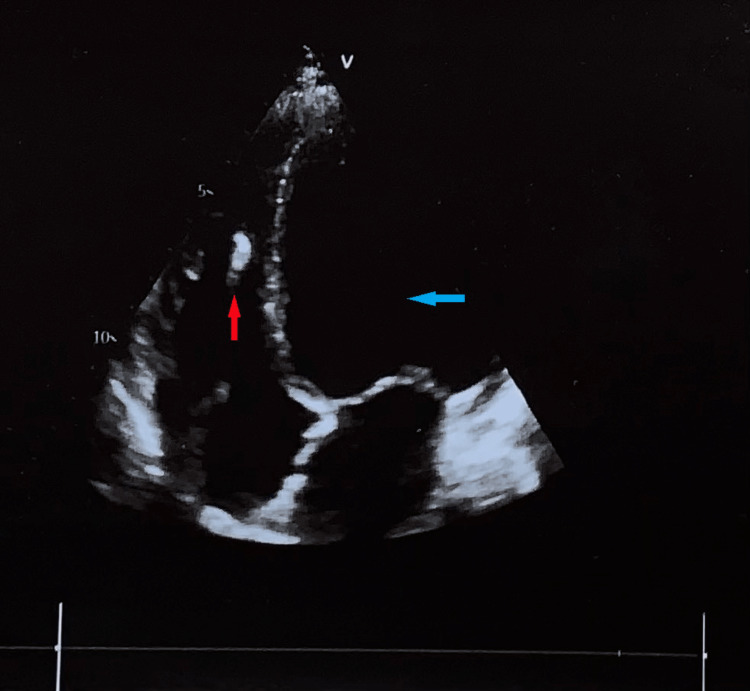
Transthoracic 2D ECHO, apical 4-chamber view showing dilated left ventricle (blue arrow). The ICD lead is visible (red arrow).

**Figure 3:**
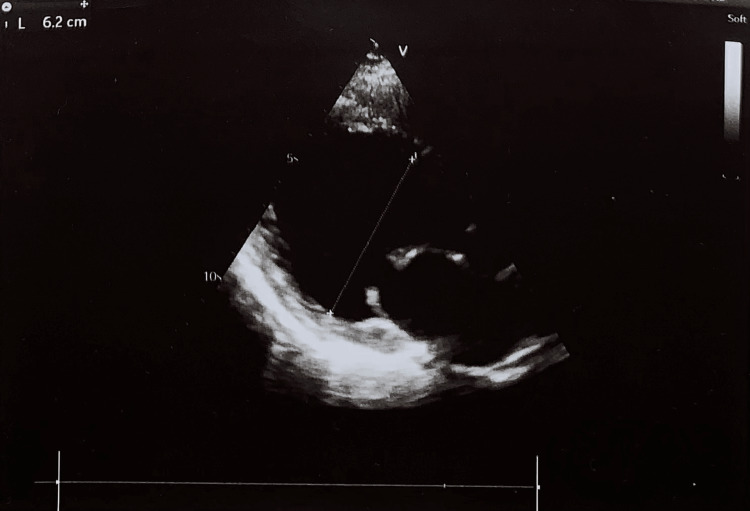
Transthoracic 2D ECHO, parasternal long-axis view showing left ventricle internal dimension in diastole of 62 mm

The Transthoracic echocardiography showed left ventricular dilated cardiomyopathy (DCM) (Figure 2) with a mild global hypokinesis of the left ventricle and moderate mitral regurgitation. Diminished systolic function with a left ventricle ejection fraction (LVEF) at 44.5%; in comparison, LVEF in 2015 was at 53%. LV internal dimension in diastole (LVIDD) in 2015 was 54 mm, while 62 mm in 2022 (Figure 3).

Subsequently, the patient was discharged with recommendations for regular outpatient cardiac follow-ups, and an angiotensin-converting enzyme (ACE) inhibitor (zofenopril 7.5 mg once daily) and iron supplementation were added to her previous regimen (metoprolol succinate 25 mg once daily).

## Discussion

In this paper, we present a case of DCM development in a young woman who, six years earlier, went through bilateral CSD because of symptomatic CPVT. To our knowledge, CPVT usually occurs in young patients who do not present any structural heart disease. However, previous studies indicate that DCM may be a long-term complication of CPVT. Existing research has established that left ventricular systolic dysfunction (LVSD) starts with an asymptomatic period, then the gradual progression of disease outweighs heart compensation possibilities and structural heart changes become apparent. For this reason, systematic ECHO screening for features of DCM development is recommended in patients who have a history of CPVT [2]. The LVSD most commonly can be the result of coronary artery disease (CAD), dilated cardiomyopathy, hypertension, infections, toxins, and valvular heart disease [6]. In our case, CAD was ruled out because coronary angiography and ECHO did not indicate regional left ventricular wall motion abnormalities or thickening of the wall during systole. Hypertension seemed less likely given normotensive during most of the hospital course. Small degree mitral regurgitation was recognized on ECHO, which was attributed to being the result of developing DCM rather than being the primary cause of LVSD. There was no past history of severe infections or cardiotoxic drugs. Further review of the patient's family history was significant for the sudden cardiac death of her sibling. A familial autosomal dominant pattern has been identified in 20 - 35% of DCM cases [7]. Genetic workup was limited in this case, for this reason, we cannot rule in or rule out the familial etiology of DCM.

During hospitalization, ECHO was performed and DCM was diagnosed by the presence of a dilated left ventricle and LVSD. The measurement of the left ventricle internal dimension in diastole (LVIDD) is used to recognize significant left ventricle dilation. The American Society of Echocardiography criteria were used to classify the degree of LVIDD. Based on mentioned criteria LVIDD can be: normal (men: 42-59 mm; women: 39-53 mm), mildly dilated (men: 60-63 mm; women: 54-57 mm), moderately dilated (men: 64-68 mm; women: 58-61 mm), or severely dilated (men: ≥69 mm; women: ≥62 mm) [8]. Early findings of a dilated ventricle with mitral regurgitation and a comparison of these data with her previous records from 2015 helped establish the most accurate diagnosis. In the case of our now 36-years old patient, her LV diameter was measured in ECHO in 2015 at 54 mm (mildly dilated), while six years later LV diameter increased to 62 mm (severely dilated). Similarly, her LVEF decreased from 53% in 2015 to 44.5% in 2021. LVEF 44.5% indicates heart failure with a mildly reduced ejection fraction (HFmrEF), though at the moment of diagnosis patient’s NT-pro-BNP level was within normal limits. The changes described above represent the early stages of the progression of DCM and heart failure (HF) development [9].

The treatment goals are focused on improving symptoms, blood circulation, and preventing further heart damage. Existing studies have provided important information on the pharmacological management of DCM. Management of heart failure with mildly reduced ejection fraction includes class I: diuretics, class IIa: SGLT2 inhibitors, and class IIb: angiotensin receptor-neprilysin inhibitor (ARNI)/ACE/ angiotensin II receptor blocker (ARB), beta-blockers, and mineralocorticoid receptor antagonist (MRA) [9].

Our patient also presented with iron deficiency microcytic hypochromic anemia, which could exacerbate her symptoms of fatigue and reduced ability to exercise and made her show up at the doctor’s office earlier. Then she was referred to our hospital for a broader diagnostic approach, and a diagnosis of DCM was established. The treatment was changed, and her current regimen includes metoprolol succinate 25 mg once daily, an ACE inhibitor (zofenopril 7.5 mg once daily), and iron supplementation. For patients with CPVT, we recommend regular follow-up visits at the cardiologist’s office and screening ECHO for diagnosis of DCM.

## Conclusions

This case report shows a rare condition of development of dilated cardiomyopathy in a young woman with a history of video endoscopic bilateral cardiac sympathetic denervation because of catecholaminergic polymorphic ventricular tachycardia. CPVT is a life-threatening inherited disorder, defined as adrenergically induced episodes of VT, presenting as stress-induced syncope, which can transform easily into VF, and consequently lead to SCD. Our patient had a severe manifestation of CPVT, which besides ICD implantation, required also bilateral CSD. The presented case revealed that CPVT may contribute to the gradual development of DCM. Management of rare cases of patients with CPVT who develop DCM is challenging. Early recognition of DCM development allows the application of the appropriate treatment, which can slow down DCM progression. We recommend regular follow-up visits at the cardiologist’s office and screening ECHO for diagnosis of DCM among patients with CPVT. Additionally, more research should be performed to illustrate the association between CPVT and DCM development.
